# Wild type cardiac amyloidosis: is it time to order a nuclear technetium pyrophosphate SPECT imaging study?

**DOI:** 10.1007/s10554-022-02692-y

**Published:** 2022-07-15

**Authors:** Christine P. Shen, Christopher T. Vanichsarn, Amitabh C. Pandey, Kristen Billick, David S. Rubenson, Rajeev C. Mohan, James Thomas Heywood, Ajay V. Srivastava

**Affiliations:** 1 Division of Cardiology, Scripps Clinic, 9898 Genesee Ave, AMP-300, La Jolla, CA USA; 2 Scripps Research Translational Institute, Scripps Research, La Jolla, CA USA

**Keywords:** Technetium99-m pyrophosphate, Transthyretin, Cardiac amyloidosis, Relative apical sparing ratio

## Abstract

Transthyretin (ATTR) amyloidosis is a debilitating systemic disease often associated with symptomatic cardiac involvement. Diagnosis has dramatically changed with the advent of Technetium-99 m pyrophosphate (Tc-PYP) single-photon emission computed tomography (SPECT). With the ability to diagnose ATTR amyloidosis noninvasively and offer newer therapies, it is increasingly important to identify which patients should be referred for this testing. Relative apical sparing of longitudinal strain on echocardiogram can be potentially used to screen such patients. We sought to describe electrocardiogram (ECG) and echocardiogram (TTE) findings, including relative apical sparing of longitudinal strain, in ATTR amyloidosis patients diagnosed non-invasively with ^99m^Tc-PYP imaging. This was a single-center, retrospective study with 64 patients who underwent ^99m^Tc-PYP imaging between June 2016 and February 2019. Relative apical longitudinal strain was calculated from left ventricular longitudinal strain (LV LS) values. No ECG parameters were meaningfully associated with of ^99 m^ Tc-PYP positive patients. LV mass index (p = 0.001), IVSd (p < 0.001), and LVPWd (< 0.001) demonstrated a highly significant difference between positive and negative ^99m^Tc-PYP groups. ^99m^Tc-PYP positive patients had a higher relative apical sparing of LV LS (p < 0.001), and notably, no ^99m^Tc-PYP negative patient had a ratio > 1.0. The finding of relative apical sparing of longitudinal strain can reliably guide clinicians in triaging which patients to consider ordering ^99m^Tc-PYP imaging for the noninvasive diagnosis of wild type cardiac amyloidosis. A patient with clinically suggestive features and an LV LS relative apical sparing ratio > 0.8 can be considered for ^99m^Tc-PYP imaging to evaluate for ATTR cardiac amyloidosis.

## Introduction

Transthyretin (ATTR) cardiac amyloidosis (CA) is an infiltrative cardiomyopathy defined by myocardial deposition of insoluble amyloid fibrils [1]. There are two distinct etiologic subtypes of ATTR: One, hereditary ATTR disease caused by variant gene mutations, and two, acquired wild-type or senile ATTR, in which misfolded plasma ATTR proteins aggregate and precipitate in the extracellular matrix of the heart [1, 2]. Historically, due to a combination of a lack of noninvasive diagnostic modalities and no direct treatment options, ATTR CA has been under-diagnosed and has been associated with a very poor prognosis, with median survival in untreated patients ranging from 2.5 to 3.6 years after diagnosis [3–5].

ATTR CA has previously been thought to be a rare entity, with a diagnosis of 70–86 per million of wild-type ATTR CA in an observational study [6]. However, autopsy studies suggest this is grossly underrepresenting of the true demographics, as 25% of patients aged 85 and over had ATTR CA on autopsy [7]. This discrepancy is likely attributable to the historical need for histologic confirmation via endomyocardial biopsy, an invasive procedure fraught with both complications and potential sampling error and the lack of supportive biomarkers (in contrast to light-chain amyloidosis) [8]. ^99m^Technetium-labeled pyrophosphate (Tc-PYP) scans display a specific affinity to ATTR amyloid deposits in the heart and have emerged as a highly specific non-invasive test for the diagnosis of ATTR CA when compared with biopsy [9–11]. When combined with negative monoclonal protein studies, the specificity and positive predictive value of ^99m^Tc-PYP scans for ATTR CA is 100%, and it is cost-effective compared with heart biopsy [8, 12]. The mortality benefit of transthyretin stabilizers such as tafamidis underscores the benefit of early diagnosis with non-invasive imaging [3]. Now, in patients without monoclonal proteins, expert consensus recommendations support the non-invasive diagnosis of ATTR CA with ^99m^Tc-PYP imaging [13].

This makes the ^99m^Tc-PYP scan a key diagnostic tool that would change the landscape of ATTR CA and broaden its diagnoses to where patients first present, the primary care clinician’s office. By using an algorithmic approach in ordering ^99m^Tc-PYP imaging, patients stand to gain tremendously by the upstream care and timing to diagnosis. In evaluating a patient for ^99m^Tc-PYP imaging, there are electrocardiographic (ECG) and echocardiographic (TTE) parameters identified as “red flags” for ATTR CA [14]. In particular, one echocardiographic finding, relative apical sparing of longitudinal strain, has been shown to have high sensitivity and specificity [15]. With the transition to non-invasive diagnosis of ATTR CA, we sought to reevaluate classic ECG and TTE parameters, including LS, and their correlation with ^99m^Tc-PYP imaging results to help guide clinicians.

## Materials and methods

A single-center retrospective cohort study of patients who were referred to Heart Failure specialists between June 2016 and February 2019 was conducted. Consecutive patients were reviewed and included in the study if they had heart failure without established etiology, New York Heart Association classification II–IV symptoms, negative laboratory work-up for AL amyloid (urine and protein electrophoresis, immunofixation electrophoresis, serum free light chain assay), and underwent a ^99m^Tc-PYP scan based on clinical suspicion for amyloidosis. Clinical, laboratory, and imaging data were acquired from the electronic medical record. Data were obtained from the closest ECG and TTE obtained prior to ^99m^Tc-PYP scanning. The study was approved by the Institutional Review Board.

### 99 m-Technetium pyrophosphate (Tc-PYP) criteria

SPECT imaging with ^99m^Tc-PYP was performed with a dual-head Philips Precedence SPECT/CT camera (Philips Healthcare, Guildford, United Kingdom). Patients received 15–25 mCi of ^99m^Tc-PYP intravenously, and anterior, lateral, and left anterior oblique planar views were obtained at one hour over 8-min durations. ^99m^Tc-PYP is readily available from commercial radiopharmaceutical distributors (TechneScan PYPTM, Mallinkcrodt, St. Louis, MO) [16, 17].

**Fig. 1:**
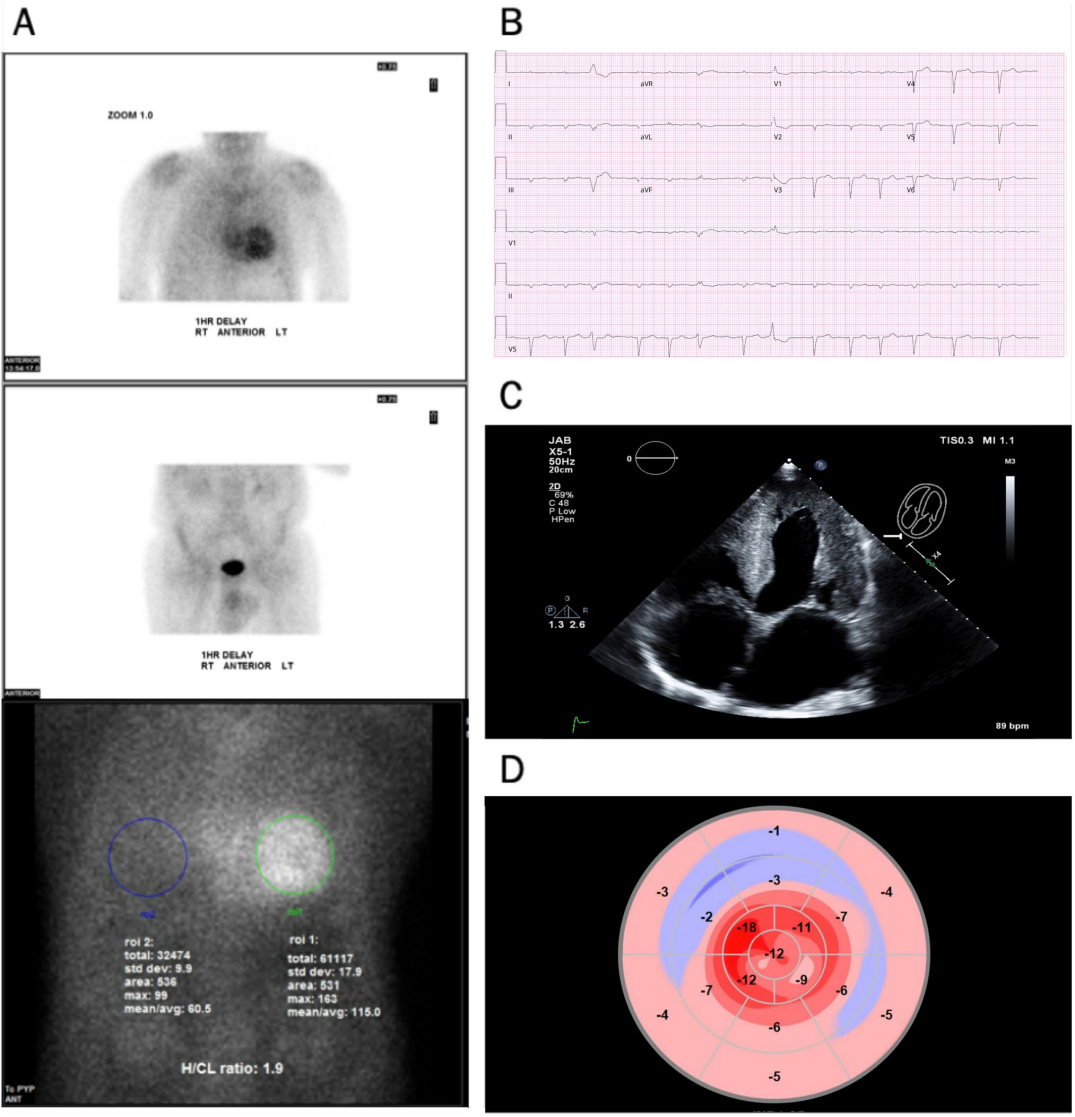
Positive ATTR Cardiac Amyloid Patient **A** ^99m^Tc-PYP scintigraphy scans demonstrating greater myocardial tracer uptake compared to bone as well as an elevated heart to contralateral lung (H/CL) ratio of 1.8. **B** Electrocardiogram with underlying rhythm of atrial fibrillation and low voltage pattern. **C** Transthoracic echocardiogram apical 4-chamber view with evidence of LV hypertrophy, thickened RV free wall, granular sparkling appearance, dilated left and right atrium. **D** Left ventricular longitudinal strain with relative apical sparing in a ‘bullseye’ pattern

SPECT imaging was used for visual interpretation and quantification of the degree of myocardial uptake by heart to lung ratio and comparison to rib uptake. In our study, we used the semi-quantitative method for the degree of myocardial uptake, using visual comparison to bone uptake at 3 h [17]. A positive ^99m^Tc-PYP scan was based on expert radiologic interpretation with a visual grade ≥ 2 and a heart-to-contralateral ratio > 1.5 (Fig. 1).

### Electrocardiogram variables

ECG analysis and measurements included: heart rate, sinus rhythm, ventricular-paced rhythm, low voltage (defined as limb lead QRS voltage < 5 mm, precordial lead QRS voltage < 10 mm), pseudoinfarct pattern (defined as pathologic Q waves > 1/4 R amplitude or QS waves in 2 consecutive leads in the absence of previous ischemic heart disease, LBBB, RBBB), LV hypertrophy (using Sokolow-Lyon criteria S wave in V1 plus R wave in V5 or V6 > 35 mm), and the presence of a bundle branch block. ECGs were interpreted by board-certified cardiologists in a blinded fashion.

### Transthoracic echocardiogram parameters

Blinded TTE measurements were obtained in accordance with the American Society of Echocardiography by board-certified cardiologists. TTE were obtained using Philips system (Phillips Medical Systems, Andover, MA, USA), and QLAB was used for global longitudinal strain. Image analysis included: LV ejection fraction (LVEF) by modified Simpson’s biplane method, LV end-diastolic volume index (LVEDV), LV mass index, interventricular septal thickness (IVSd) measured in end-diastole, LV internal diameter (LVIDd) measured in end-diastole, LV posterior wall thickness (LVPWd) measured in end-diastole, relative wall thickness (RWT), left atrial (LA) volume index, right atrial (RA) volume index, LV outflow tract velocity time integral (LVOT VTI), RV basal diameter (RVd1), RV mid diameter (RVd2), tricuspid annular plane systolic excursion (TAPSE), RV tissue Doppler systolic velocity (RV S′), mitral inflow early E velocities, medial and lateral mitral annular tissue Doppler velocities (e′), average E/e′, pulmonary artery systolic pressure (PASP), and mean pulmonary artery pressure (mPAP). Global longitudinal strain (GLS), 2-chamber LS, 3-chamber LS, and 4-chamber LS analysis was performed offline in a blinded fashion. Relative apical LS (RAS) was calculated using the equation [mean apical-LS/(mean basal-LS + mean mid-LS)] [15].

### Statistical analyses

All continuous variables were expressed as mean ± SD standard deviation and statistical analysis performed using Mann–Whitney *U* test. Categorical variables were expressed as n (%) and statistical differences were calculated by Fisher’s exact test. All statistical analysis was performed using GraphPad Prism 8.0.2 software (GraphPad Prism, La Jolla, CA). All hypothesis tests were two-sided. A p-value < 0.05 was used for statistical significance.

## Results

A total of 64 heart failure NYHA class II–IV patients without established etiology with negative AL amyloid biomarkers were referred for ^99m^Tc-PYP scanning from June 2016 to February 2019. Of the 64 patients: 31 patients had positive scans, 30 patients had negative scans, and 3 patients had an equivocal semi-quantitative score and were excluded from the data analysis. Based on the clinical picture according to guidelines, the patients in this study with positive scans were diagnosed with ATTR CA [13].

Baseline demographic and clinical characteristics are shown, with a mean age of 76.5 (SD ± 10.0) years (Table 1). Patients with positive scans were much more likely to be men (94% vs. 53%, p < 0.001) and have a prior diagnosis of atrial fibrillation (74% vs. 47%, p = 0.04).

**Table 1:** Baseline demographics of 99mTc-PYP negative and positive patients

Characteristic	Tc-PYP -	Tc-PYP +	p-value
Age (years)	73.3 SD 11.46	79.6 SD 7.37	0.01
Sex (male)	53% (16/30)	94% (29/31)	< 0.001
BMI (kg/m^2^)	25.3 SD 7.86	26.0 SD 3.09	0.65
Hypertension	83% (25/30)	87% (27/31)	0.73
AV disease (≥ mod AS or s/p AVR)	10% (3/30)	19% (6/31)	0.47
Coronary artery disease	37% (11/30)	48% (15/31)	0.44
Atrial fibrillation	47% (14/30)	74% (23/31)	0.04
Diabetes mellitus	40% (12/30)	19% (6/31)	0.10
CKD stage (mean)	1.73 SD 1.86	1.35 SD 1.56	0.39
Hemodialysis	10% (3/30)	3% (1/31)	0.35
Pacemaker/ICD	17% (5/30)	42% (13/31)	0.05
Neuropathy	53% (16/30)	35% (11/31)	0.20
Carpal tunnel syndrome	23% (7/30)	45% (14/31)	0.11

Descriptive data are presented as frequency % (patients with the characteristic / total patients in the group). Continuous variables are expressed as mean SD standard deviation

*AS* aortic stenosis, *AV* aortic valve, *AVR* aortic valve replacement, *CKD* chronic kidney disease, *ICD* implantable cardioverter defibrillator, *s/p* status post

Baseline ECG findings were available for all patients (Table 2). Four patients in the ^99m^Tc-PYP negative group, and 11 patients in the ^99m^Tc-PYP positive group were excluded from the analysis of pseudoinfarct pattern and low voltage assessment due to underlying ventricular-paced rhythm or bundle branch block morphology. Sinus rhythm was found in eight (26%) patients in the ^99m^Tc-PYP positive group versus 18 (60%) in the ^99m^Tc-PYP negative group (p = 0.01). There was no significant difference between the two groups in findings of low voltage ECG (p = 0.57), low voltage with pseudoinfarct pattern (p = 0.18), or bundle branch block morphology (left p > 0.999, right p = 0.21).

**Table 2:** Electrocardiographic variable of 99mTc-PYPnegative and positive patients

Variables	Tc-PYP-	Tc-PYP +	p-value
Heart rate	73.50 SD 11.57	69.68 SD 12.79	0.37
Sinus rhythm	60% (18/30)	26% (8/31)	0.01
Atrial fibrillation/flutter	23% (7/30)	39% (12/31)	0.27
Ventricular paced rhythm	13% (4/30)	32% (10/31)	0.13
Pseudoinfarct pattern	19% (5/26)	40% (8/20)	0.19
Low voltage	4% (1/26)	10% (2/20)	0.57
Low voltage and pseudoinfarct pattern	0% (0/26)	10% (2/20)	0.18
LVH	8% (2/26)	0% (0/20)	0.50
LBBB	0% (0/26)	0% (0/20)	0.99
RBBB	8% (2/26)	25% (5/20)	0.21

*LBBB* left bundle branch block, *LVH* left ventricular hypertrophy, *RBBB* right bundle branch block

Key TTE features show mean LVEF was 61.5% (SD 13.5, range 38–80%) in the ^99m^Tc-PYP negative group versus 49.7% (SD 14.4, range 14–78%) in the ^99m^Tc-PYP positive group (p = 0.002) (Table 3). Medial e’ velocity was significantly reduced in the ^99m^Tc-PYP positive patients (4.14 SD 1.11 vs. 5.55 SD 1.81, p = 0.004), but there were no differences between the two groups in lateral e’ (7.23 SD 3.05 in 99mTc-PYPnegative group versus 5.73 SD 1.78 in ^99m^Tc-PYP positive group, p = 0.06) and average E/e’ parameters (5.55 SD 1.84 in ^99m^Tc-PYP negative group versus 4.14 SD 1.11 in ^99m^Tc-PYP positive group, p = 0.004). Both RV function parameters were statistically reduced (TAPSE, RV S’ velocity) (p value of each) (Table 3).

**Table 3:** Echocardiographic parameters of 99mTc-PYPnegative and positive patients

	Parameters	Tc-PYP−	Tc-PYP +	p-value
LV function parameters	Ejection fraction (%)	61.5 SD 13.5	49.7 SD 14.4	0.002
	Mitral E wave (cm/s)	98.0 SD 40.0	85.5 SD 28.1	0.23
	Lateral e’ (cm/s)	7.23 SD 3.05	5.73 SD 1.78	0.06
	Medial e’ (cm/s)	5.55 SD 1.84	4.14 SD 1.11	0.004
	Average E/e’ ratio	16.9 SD 7.36	18.9 SD 7.86	0.13
RV function parameters	TAPSE (cm)	2.1 SD 0 .68	1.59 SD 0.55	0.02
	RV S’ (cm/s)	11.7 SD 3.25	8.84 SD 2.69	< 0.001
Chamber size/dimensions	LVEDV index (ml/m^2^)	50.2 SD 22.2	46.1 SD 15.9	0.49
	LV mass index (g/m^2^)	125 SD 36.1	166 SD 50.5	0.001
	IVSd (cm)	1.37 SD 0.32	1.80 SD 0.44	< 0.001
	LVIDd (cm)	4.53 SD 0.85	4.25 SD 0.62	0.46
	LVPWd (cm)	1.32 SD 0.44	1.68 SD 0.37	< 0.001
	RWT	0.57 SD 0.18	0.80 SD 0.25	< 0.001
	LA volume index (ml/m^2^)	50.3 SD 20.2	51.7 SD 16.5	0.55
	RA volume index (ml/m^2^)	47.8 SD 34.5	49.0 SD 20.8	0.36
	RVd1 (mm)	4.01 SD 0.91	4.08 SD 0.77	0.62
	RVd2 (mm)	2.92 SD 0.92	2.65 SD 0.81	0.40
Hemodynamic parameters	PASP (mmHg)	48.1 SD 16.3	39.3 SD 7.40	0.08
	mPAP (mmHg)	34.7 SD 11.9	38.1 SD 5.84	0.06
	LVOT VTI (cm)	19.9 SD 7.50	15.2 SD 4.78	0.01
LV longitudinal strain	GLS	− 17.0 SD 4.47	− 13.8 SD 3.80	0.01
	LS 2-chamber	− 17.2 SD 5.31	− 13.9 SD 4.08	0.03
	LS 3-chamber	− 17.0 SD 4.24	− 13.6 SD 3.82	0.003
	LS 4-chamber	− 16.8 SD 5.20	− 13.4 SD 4.35	0.01
	Relative Apical LS	0.72 SD 0.11	0.94 SD 0.16	< 0.001

*GLS* global longitudinal strain, *IVSd* interventricular septal thickness in diastole, *LA* left atrium, *LV* left ventricle, *LVEDV* left ventricle end-diastolic volume, *LVOT* left ventricle outflow tract, *LVID* left ventricle internal diameter, *LVPWd* left ventricle posterior wall thickness in diastole, *mPAP* mean pulmonary artery pressure, *PASP* pulmonary artery systolic pressure, *RA* right atrium, *RV* right ventricle, *RVd* right ventricle diameter, *RWT* relative wall thickness, *TAPSE* tricuspid annular plane systolic excursion, *VTI* velocity time integral

Significant increases were seen in the ^99m^Tc-PYP positive group across multiple chamber size/dimensions, including LV mass index (166 SD 50.5 vs. 125 SD 36.1, p = 0.001), IVSd (1.80 SD 0.44 vs. 1.37 SD 0.32, p < 0.001), LVPWd (1.68 SD 0.37 vs. 1.32 SD 0.44, p < 0.001), and RWT (0.80 vs. 0.57, p < 0.001). No changes were seen between LA and RA volume indexes, as well as mid- and basal-RV diameters (Table 3).

Evaluation of hemodynamic parameters including PASP and mPAP were not statistically different between the two groups (Table 3). However, LVOT VTI was significant reduced in the ^99m^Tc-PYP positive patients (p = 0.01).

**Fig. 2:**
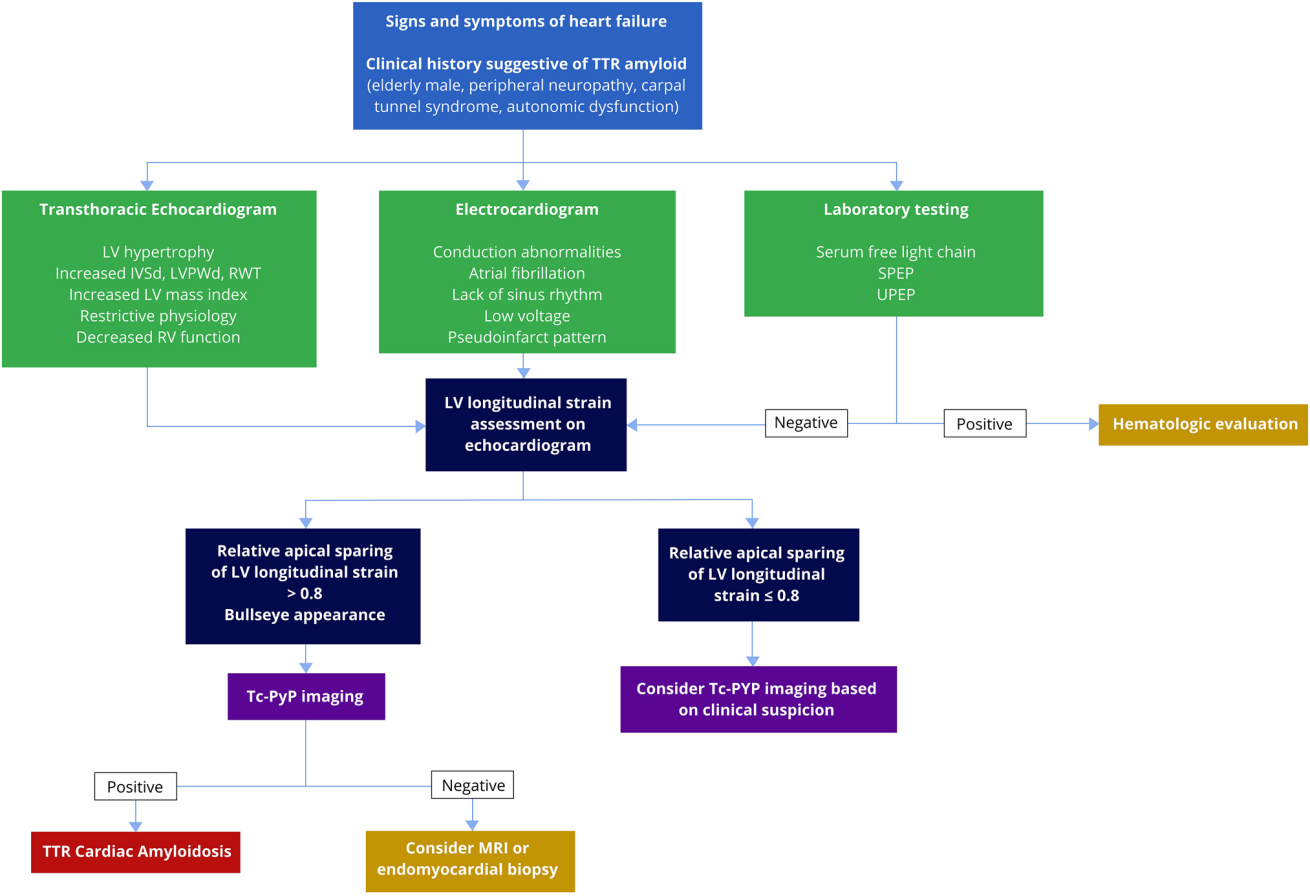
Proposed Diagnostic Algorithm for ATTR Cardiac Amyloid The algorithm starts with a patient with signs and symptoms of heart failure, particularly with a clinical history suggestive of ATTR CA. The patient should undergo further testing with transthoracic echocardiogram, electrocardiogram, and laboratory testing. Echocardiogram and electrocardiogram findings characteristic of ATTR CA, in conjunction with negative laboratory testing for light chain amyloidosis, should prompt a retrospective LS assessment on echocardiogram. We choose the RAS cut-off of 0.8, above which patients should move forward with ^99m^Tc-PYP imaging to diagnose ATTR CA. If at or below 0.8, ^99m^Tc-PYP imaging can be considered based on clinical suspicion

Additionally, left ventricular GLS was significantly lower in the ^99m^Tc-PYP positive group (− 13.8 vs. − 17.0, p = 0.01), and individually across all three LV views (Table 3). Evaluation of RAS showed a calculated RAS ratio of 0.72 (SD 0.11, range 0.56–0.88) in the ^99m^Tc-PYP negative group compared to 0.94 (SD 0.16, range 0.54–1.18) in the positive group (p < 0.001). As illustrated in Table 4, using a RAS ratio cut-off of ≥ 1.0 was 100% specific with a 100% PPV for identifying ^99m^Tc-PYP positive patients, though test sensitivity was very low at 32%. Reducing the RAS ratio cut-off to ≥ 0.8 improved test sensitivity up to 87.1% at the expense of lowering specificity and PPV to 76.7% and 79.4%, respectively. With the findings from our study, we propose a clinical algorithm for patients with heart failure to undergo ^99m^Tc-PYP imaging (Fig. 2) to diagnose ATTR CA.

**Table 4:** Sensitivity, specificity, and positive and negative predictive values for variable relative apical sparing cut-offs

	Sensitivity	Specificity	Positive predictive value	Negative predictive value
RAS ≥ 1	33.3% (16.7–51.4%)	100% (88.4–100%)	100%	58.8% (52.8–64.6%)
RAS ≥ 0.9	54.8% (36.0–72.7%)	96.7% (82.8–99.9%)	94.4% (70.7–99.2%)	67.4% (58.3–75.4%)
RAS ≥ 0.85	77.4% (58.9–90.4%)	86.7% (69.3–96.2%)	85.7% (70.3–93.8%)	78.8% (65.6–87.9%)
RAS ≥ 0.8	87.1% (70.2–96.4%)	76.7% (57.7–90.1%)	79.4% (66.5–88.2%)	85.2% (69.3–93.6%)

*RAS* relative apical sparing

## Discussion

Our study provides detailed analyses on clinical, electrical, and morphologic and functional parameters seen in ATTR CA diagnosed by ^99m^Tc-PYP scintigraphy imaging. Using a noninvasive imaging-based protocol, the present study identifies a subset of patients with ATTR CA who were more likely to be male and with a history of carpal tunnel syndrome. Additionally, ^99m^Tc-PYP positive patients were more likely to be older with coronary artery disease and higher rates of pacemaker and ICD implantations.

While the ECG has long been considered a helpful initial screening test, with reported typical ECG findings of low voltage QRS, pseudoinfarct pattern, and atrial fibrillation [14], often, ECG findings tend to be nonspecific or completely uninterpretable. The actual reported prevalence of these findings in patients with confirmed ATTR CA is highly variable, with low voltage QRS ranging from 7 to 42%, and pseudoinfarct pattern from 10 to 38% [4, 18–21]. Apart from being nonspecific findings in isolation, nearly half of our ATTR amyloid patients (15 out of 31, 48%) had ECG findings (V-paced rhythm, bundle branch block) that precluded any interpretation of QRS voltage, pseudoinfarct pattern, or LVH. Apart from a lack of sinus rhythm, no ECG parameters were associated with ^99m^Tc-PYP positive patients. This suggests that ECG has significant limitations in screening for ATTR CA and should not be used.

Our study corroborates previously reported TTE findings in the literature highlighting changes in chamber size/dimensions and LV systolic and diastolic function patterns seen in ATTR amyloid cardiomyopathy [22]. ^99m^Tc-PYP positive patients had a mildly reduced comparative LVEF, which may be representative of progressive deterioration of LV function in our elderly population (average age 76.5 years), as well as more restrictive filling pattern as demonstrated by significantly reduced medial e’ velocities [13, 23]. Even though ATTR negative patients had baseline measurements consistent with concentric hypertrophy, ATTR positive patients displayed significantly increased dimensions across the board, including LV mass index, IVSd, LVPWd, and RWT. Although these findings appear to be consistent and highly reproducible across multiple studies, they individually lack specificity in differentiating ATTR CA from common mimickers [20, 24].

The additive value of performing LS in this population of LVH is highlighted in this study. Overall GLS was significantly reduced in the ATTR positive patients (p < 0.001). More importantly, quantifying a relative apical sparing pattern cut-off of 0.8 demonstrated an 87% sensitivity and 78% specificity in discriminating ATTR CA, optimizing sensitivity for a screening test for a life-threatening but treatable disease [25]. This makes it a highly useful differentiating parameter that increases the clinical suspicion for ATTR CA and can be used in an algorithmic approach to guide clinicians on when to order nuclear testing with ^99m^Tc-PYP imaging (Fig. 2). We propose an algorithm that starts with a patient with history of heart failure, particularly one with carpal tunnel or neuropathy. The patient should undergo further testing with TTE, EKG, and laboratory testing. Echocardiogram and electrocardiogram findings characteristic of ATTR CA, in conjunction with negative laboratory testing for light chain amyloidosis, should prompt a LS assessment on echocardiogram. We choose the RAS cut-off of 0.8, above which there is an increased suggestion of ATTR CA, and patients should move forward with nuclear testing with ^99m^Tc-PYP imaging to diagnose ATTR CA. If at or below 0.8, ^99m^Tc-PYP imaging can still be considered based on the pre-test clinical suspicion.

## Study limitations

This is an observational, retrospective study performed at a single center. The sample size limits the power of the study but given that CA has been thought to be a rare disease and is only recently being diagnosed with increasing frequency this is not totally surprising. Our study numbers are comparable to previously published studies. Evaluating for spatial correspondence between SPECT imaging and echocardiography can provide additional insight into its diagnostic value. While our patients did not undergo confirmatory endomyocardial biopsies, expert guidelines support non-invasive diagnosis with scintigraphy for patients without the presence of a monoclonal protein [8, 13].

## Conclusions

ATTR CA remains severely underdiagnosed. With the advent of ^99m^Tc-PYPscans as a feasible noninvasive diagnostic modality, it is imperative that clinicians outside of specialized Amyloid centers be familiar with clues on history and concomitant cardiac tests to allow upstream diagnosis and thereby increasing access to care. Taken in combination with a patient’s clinical history, specific ECG patterns, and particular TTE parameters, the routine use of LV LS and RAS calculations can help delineate at-risk patients who should undergo nuclear scintigraphy. An increased awareness layered with an algorithmic approach will allow earlier diagnosis of ATTR CA and thereby get more patients on transthyretin stabilizing therapies.
